# Supplementary material for: Splice-Junction-Based Mapping of Alternative Isoforms in the Human Proteome
Source: Cell Rep. Author manuscript; Available in PMC 2020 Jan 15. (PMC6961840; doi:10.1016/j.celrep.2019.11.026)
Supplement: 2 [file NIHMS1546469-supplement-2.zip › DF1/PXD010154/Heart/Heart_18_TTN_RVEAEPAEVTEFR.pdf]

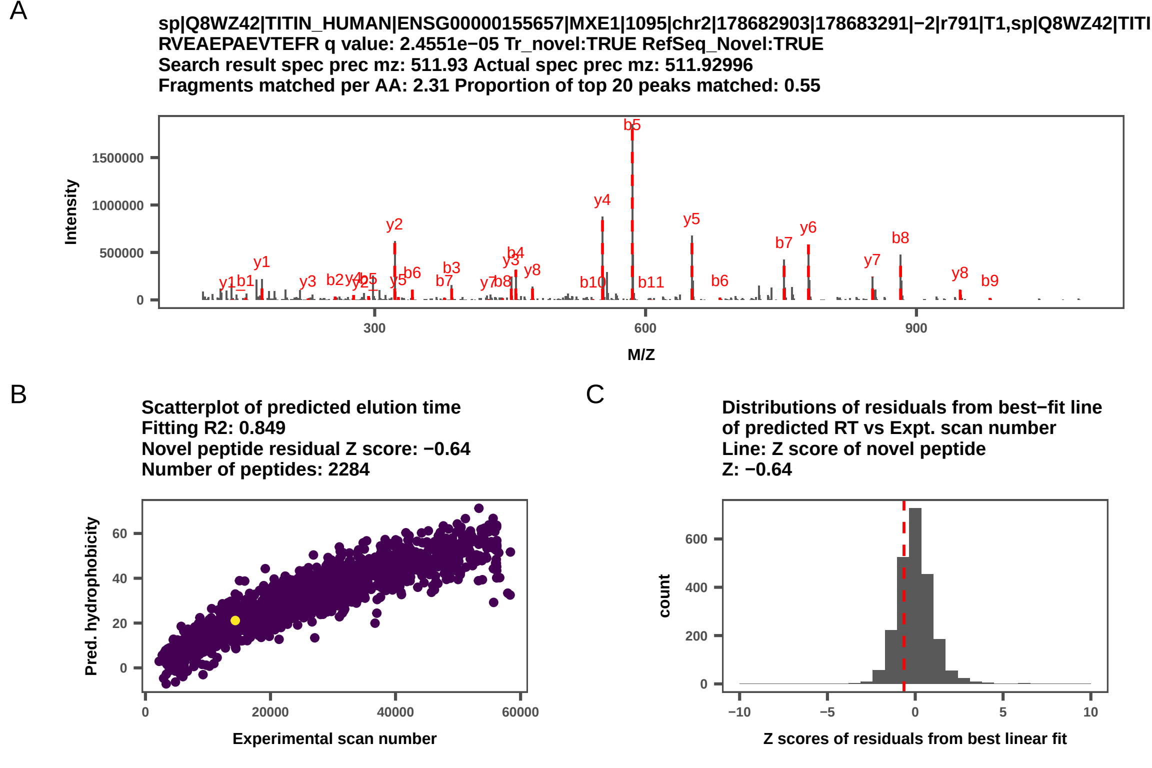
A
sp|Q8WZ42|TITIN_HUMAN|ENSG00000155657|MXE1|1095|chr2|178682903|178683291|−2|r791|T1,sp|Q8WZ42|TITI
RVEAEPAEVTEFR q value: 2.4551e−05 Tr_novel:TRUE RefSeq_Novel:TRUE
Search result spec prec mz: 511.93 Actual spec prec mz: 511.92996
Fragments matched per AA: 2.31 Proportion of top 20 peaks matched: 0.55
Intensity
M/Z
B
Scatterplot of predicted elution time
Fitting R2: 0.849
Novel peptide residual Z score: −0.64
Number of peptides: 2284
Pred. hydrophobicity
Experimental scan number
C
Distributions of residuals from best−fit line
of predicted RT vs Expt. scan number
Line: Z score of novel peptide
Z: −0.64
count
Z scores of residuals from best linear fit
